# A Longitudinal Analysis Reveals Early Activation and Late Alterations in B Cells During Primary HIV Infection in Mozambican Adults

**DOI:** 10.3389/fimmu.2020.614319

**Published:** 2021-01-15

**Authors:** Montse Jiménez, Lucía Pastor, Victor Urrea, María Luisa Rodríguez de la Concepción, Erica Parker, Laura Fuente-Soro, Chenjerai Jairoce, Inacio Mandomando, Jorge Carrillo, Denise Naniche, Julià Blanco

**Affiliations:** ^1^ AIDS Research Institute-IrsiCaixa, Institut Germans Trias i Pujol (IGTP), Hospital Germans Trias i Pujol, Universitat Autonoma de Barcelona, Badalona, Spain; ^2^ ISGlobal, Barcelona Institute for Global Health, Hospital Clínic–Universitat de Barcelona, Barcelona, Spain; ^3^ Centro de Investigação em Saúde da Manhiça (CISM), Maputo, Mozambique; ^4^ Faculty of Health and Medical Sciences, University of Western Australia, Perth, WA, Australia; ^5^ Universitat de Vic—Universitat Central de Catalunya (UVIC-UCC), Vic, Spain

**Keywords:** B-cell activation, plasmablasts, marginal zone (MZ) B cell, transitional B cell, PD-1

## Abstract

Primary HIV infection (PHI) and subsequent chronic infection alter B-cell compartment. However, longitudinal analysis defining the dynamics of B-cell alterations are still limited. We longitudinally studied B-cell subsets in individuals followed for 1 year after PHI (n = 40). Treated and untreated chronic HIV infected (n = 56) and HIV-uninfected individuals (n = 58) were recruited as reference groups at the Manhiça District in Mozambique. B cells were analyzed by multicolor flow-cytometry. Anti-HIV humoral response and plasma cytokines were assessed by ELISA or Luminex-based technology. A generalized activation of B cells induced by HIV occurs early after infection and is characterized by increases in Activated and Tissue-like memory cells, decreases in IgM-IgD- (switched) and IgM-only B cells. These alterations remain mostly stable until chronic infection and are reverted in part by ART. In contrast, other parameters followed particular dynamics: PD-1 expression in memory cells decreases progressively during the first year of infection, Transitional B cells expand at month 3–4 after infection, and Marginal zone-like B cells show a late depletion. Plasmablasts expand 2 months after infection linked to plasma viral load and anti-p24 IgG3 responses. Most of well-defined changes induced by HIV in B-cell activation and memory subsets are readily observed after PHI, lasting until ART initiation. However, subsequent changes occur after sustained viral infection. These data indicate that HIV infection impacts B cells in several waves over time, and highlight that early treatment would result in beneficial effects on the B-cell compartment.

## Introduction

HIV infection mainly targets myeloid and CD4+ T cells (1); however, the damage infringed by chronic viral replication spreads to the whole immune system, including CD8 T cells and B cells (2, 3). For T and B-cell compartments, a generalized hyper-activation status has been widely described, that specifically in B cells is characterized by an increased frequency of activated memory cells and hypergammaglobulinemia (4). A large number of studies have provided a detailed description of the impact of chronic HIV infection (CHI) on the phenotype and function of B cells [excellently reviewed in (5)], demonstrating that the homeostasis of B-cell subsets is drastically affected by CHI resulting in expansion of immature/transitional cells (6), activated memory cells, and differentiated plasmablasts (4). Perturbations of mature B cells include an impact on naïve cells, that are reduced in number and function early after HIV infection (7), and memory B cells, which seem to be the most affected subset, showing lower numbers of switched IgM-IgD- memory cells, expansion of IgM-only memory cells (8) and an aberrant Tissue-like memory (TLM) repertoire (9). Besides altering B-cell subsets, HIV infection also impairs B-cell function. The expression of the check-point receptor programmed cell-death protein 1 (PD-1) has been widely associated with B-cell dysfunction and increased levels of this marker have been described in primary HIV infection (PHI) (7) and CHI (10, 11). PD-1 may impact general (12) and specific anti-HIV humoral responses by affecting different subsets, including TLM cells, which appear to encompass an important fraction of HIV specific B cells (13). B-cell dysfunction affects both T-dependent and independent responses. The latter is controlled by the Marginal Zone-like (MZ-like) B-cell population, which is reduced in CHI individuals, although the splenic function seem to be maintained (8).

As for other viral infections, PHI induces a rapid immune response including sequentially: a prominent response of inflammatory cytokines (the “cytokine storm”) (14), followed by a rapid expansion and activation of CD8 T cells and antibody secreting cells (plasmablast) (7, 14, 15). However, the challenges of identifying PHI and the lack of longitudinal studies addressing B-cell phenotypes and function in recently HIV infected patients have resulted in a lack of information on the timing of events leading to chronic B-cell damage after HIV-infection, especially in Sub-Saharan African populations (16). Studies addressing early/acute or hyperacute HIV-infected PHI individuals indicate early alterations of B cells similar to those reported in chronic infection (7, 17); however, the impact of such early alterations on humoral responses to HIV (17) and on chronic B-cell dysfunction are still poorly defined (18, 19). Interestingly, antiretroviral treatment (ART) fails to fully normalize B-cell dysfunction (8), including lack of response to vaccines which remain impaired in treated HIV-infected individuals (20).

In this study, we provide a longitudinal characterization of different B-cell subsets over the first year after PHI in a cohort of Mozambican adults and we compare these changes with CHI individuals, either ART-naïve or treated, and HIV-uninfected subjects. Additionally, we explore the association of B-cell alterations with cytokine and humoral responses elicited against HIV. Our data suggest the occurrence of a multi-wave immunological B-cell damage, characterized by early activation associated with antiviral responses and subsequent events that extend several months after viral setpoint has been achieved.

## Material and Methods

### Study Population

The present study is a sub-analysis of a prospective cohort of primary HIV-infected adults enrolled and followed up for 12 months in the Gastro-intestinal biomarkers in Acute-HIV infected Mozambican Adults (GAMA) study (15, 21–23). The study population was enrolled between 2013 and 2014 at the Manhiça District Hospital (MDH) in Southern Mozambique (15, 22).

Participants (>18 years of age) were screened at the outpatient clinic of MDH for non-specific febrile symptoms or voluntary HIV testing. PHI participants (n = 57) were identified by negative or indeterminate rapid serology test and positive HIV-RNA test. A control population was established by randomly selecting HIV-uninfected individuals at the outpatient clinic (n = 58). Additionally, adults with documented HIV diagnosis for more than 12 months attending routine scheduled visits were enrolled as CHI patients. CHI patients were included as ART-naïve or treated, depending whether they had previously initiated treatment according to the national guidelines at recruitment (ART was provided to individuals with a CD4 T-cell count ≤350 cells/mm3 or presenting AIDS-associated diseases). A subset of PHI individuals (n = 44) were followed up for 12 months as previously described (15). Briefly, only plasma samples were available at PHI recruitment, while follow-up samples from month 2 (M2) to month 12 post infection (M12), CHI and HIV-uninfected individuals included stool and cryopreserved PBMC samples, which were used for B-cell immunophenotyping. Sample timepoints were adjusted by Fiebig stage as described (15).

After recruitment, demographic and clinical data was collected and medical consultation and HIV counseling was provided to all participants. CD4 and CD8 T-cell counts in fresh blood was determined using Trucount tubes and a FACScalibur flow cytometer. PBMCs were isolated by Ficoll density gradient and were stored in liquid nitrogen. Plasma Viral Load (VL) determination was performed as previously described (15). Additional testing for most prevalent infections in the area, including malaria, hepatitis B virus (HBV), syphilis, gastro-intestinal protozoa, bacteria, and parasites was also performed (15).

Multiplex biomarker profiling was performed for a total of 61 immune response inflammatory biomarkers in plasma samples (22, 23). In parallel, humoral responses against HIV proteins Env, Gag, and Integrase (In) were determined by Luminex based technology in plasma samples (22). The phenotype of peripheral T cells was previously described (15).

### B-Cell Immunephenotyping

Cryopreserved PBMCs were thawed at 37°C, washed in RPMI/60% and RPMI/20% of fetal bovine serum (FBS), and incubated for 1 h at 37°C in RPMI/10%FBS. PBMCs were then stained with the Fixable Viability Stain-FVS780r (APC-H7 detect, BD Biosciences) for 15 mins. After PBMCs washing in PBS/1%FBS, cells were incubated in a U-bottom 96-well plate at a density of 1.5 million/well and stained with selected 14-color panel including: anti-CD19-AF700 (Clone HIB19), anti-IgD-Pe-Cy7 (Clone IA6-2), anti-IgM-BB515 (Clone G-20-127), polyclonal goat anti-IgA-Dylight^®^649 (from Jackson Immunoresearch), CD10-BV650 (Clone H10a), CD21-PE-CF594 (Clone B-LY4), CD27-BV510 (Clone L128), CD38-BV786 (Clone HIT2), CD45-RB-PE (Clone MT4), CD86-PerCP-Cy5.5 (Clone 2331), CD279 (PD-1)-BV421 (Clone EH12.1), all from BD Biosciences unless indicated, for 15 mins. After washing twice in PBS/1% FBS, cells were fixed in PBS/1% formaldehyde, acquired in a BD LSRFortessa (BD Biosciences) cytometer using a plate HTS loader (BD Biosciences) and analyzed with FlowJo software (Tree Star). Gating strategy is described in **Supplementary Figure 1**. Lymphocyte gate was defined manually by morphological parameters excluding non-viable cells. B cells were identified as CD19+ CD21+/− cells and gated according to the expression of different markers to identify B-cell maturation stages as described in **Supplementary Figure 1**.

### Statistical Analysis

Intergroup comparisons were performed using the Fisher exact test for categorical variables and the non-parametric Kruskal-Wallis test for continuous variables, using Dunn’s test for *post-hoc* pairwise comparisons. Spearman’s correlation was used to assess correlations between continuous variables and multiple testing was further adjusted by False Discovery Rate (FDR). Relative changes (Z-score) with respect to the control (HIV-uninfected) group have been represented by a transformation of the fitted longitudinal models by subtracting the mean and dividing by the standard deviation of Control group distribution (logarithmic transformation was used for non-normal distributions). Longitudinal models for the different immunological variables were modeled by fitting smoothing-splines mixed-effects models using the “sme” package of R. To infer if there was a significant association of selected biomarkers with the time variable, polynomial time effects approximation until third degree were fitted using linear mixed-effects regression models. Best model was selected based on likelihood-ratio tests under maximum likelihood model estimations. Statistical analyses were performed using R-3.3.1 and Stata14 software.

## Results

### Characteristics of the Study Population

From the 57 individuals identified as PHI, 44 attended the follow-up visits and provided blood samples for PBMC isolation and subsequent B-cell subset analysis. Among them 21, 5, 7, and 11 were categorized into Fiebig I-III, Fiebig IV, Fiebig V, and Fiebig VI stages, respectively, and were adjusted for time since infection as described previously (15). Fifty-eight HIV-uninfected individuals were randomly selected from screened negative individuals and 56 CHI individuals were recruited at the Manhiça District Hospital, 26 of them were untreated (CHI-naïve) and 30 were on ART (CHI-ART). The demographic and clinical characteristics of the 40 PHI individuals who started follow-up, the HIV-uninfected and the CHI groups, have been previously described (15). Briefly, PHI individuals were mostly young (mean 27-year-old), females (60%), and most prevalent co-infections were HBV, intestinal infections, syphilis, and malaria [<20% of recruited individuals presented some of these infections, **Supplementary Table 1** (15)].

**Figure 1:**
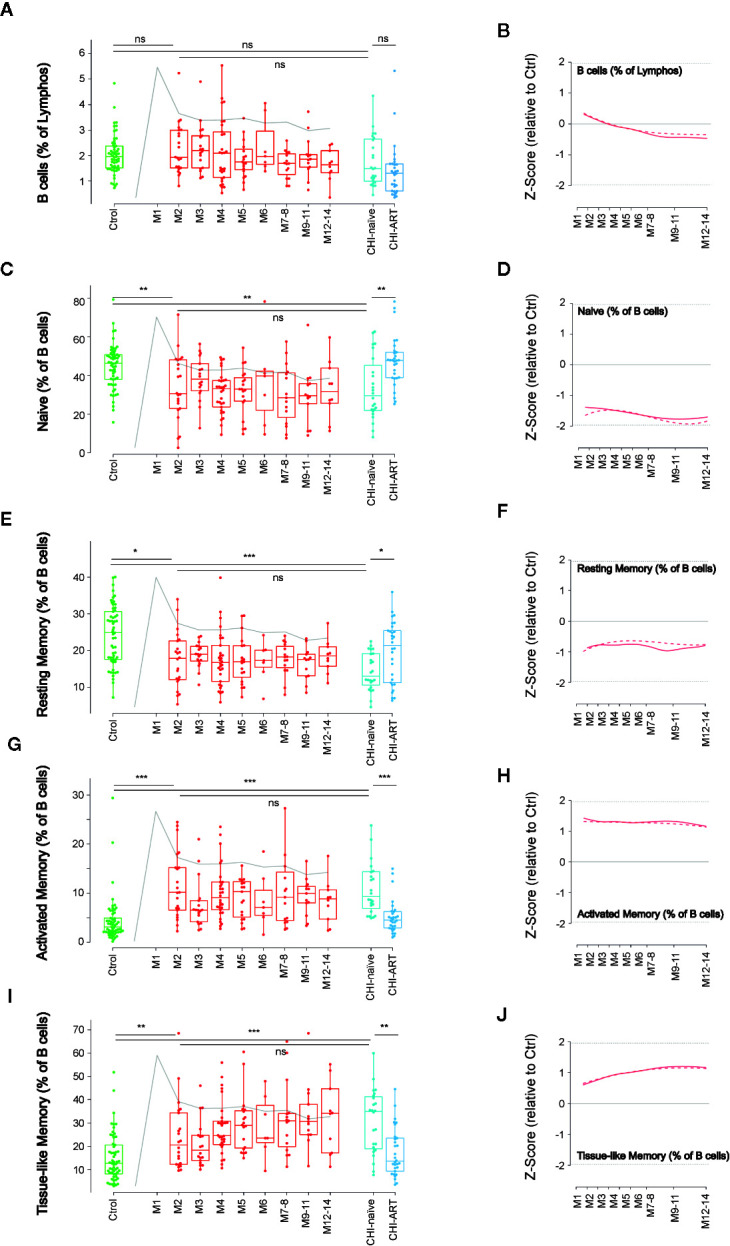
Dynamics of main B-cell subsets after HIV infection. Left panels show individual data observations, median, IQR (box), and min/max values (bars, except for outliers) across the different timepoints and study groups: HIV-uninfected (green), primary HIV infected (red, M indicates months after infection), and chronically naïve (CHI-naïve) or treated (CHI-ART) HIV infected individuals (light and dark blue respectively). Gray line represents the profile of VL dynamics for clinical reference. Right panels show the modeled dynamics as Z-score values for PHI individuals over HIV-uninfected individuals. Solid lines show non-parametric models, while dotted lines indicate the best fitting for polynomial time effects regression approximation. The frequency of total B cells **(A**, **B)** and the different subsets defined by CD21 and CD27 markers **(C**–**J)** is shown as percentage. ns non significant; *p < 0.05; **p < 0.01; ***p < 0.005.

After adjusting for time since infection according to Fiebig stage, peak VL was identified at 1 month after infection, M1) with a median VL value of 6.9 RNA Log10 copies/ml (IQR 6.2–7.5), rapidly stabilizing afterwards (**Figure 1A**, gray line) (15). CD4 T-cell counts were significantly lower in PHI as compared to HIV-uninfected individuals (median 565 cells/µl at M2 after infection and 855 cells/µl, respectively, p < 0.001) and remained stable over the first year of infection (15). CD8 T cells showed maximal expansion at M2 and slowly decreased until M5-6 to achieve the immunological setpoint (15).

### Dynamics of B-Cell Subsets and Association With Viral Pathogenesis

We initially analyzed the frequency of B cells (CD19+) and their functionally distinct subsets defined by CD21 and CD27 expression: Naïve cells (CD21+CD27−), resting memory cells (CD21+CD27+), activate memory cells (CD21–CD27+), and TLM cells (CD21−CD27−). The dynamics of the different populations along the first year of HIV-infection was analyzed using non-parametric and linear regression modeling as described (15).

No significant changes in the frequency of B cells were observed over PHI (**Figures 1A, B**). However, frequencies of the main B-cell subsets rapidly changed after infection compared to HIV-uninfected individuals, with a significant reduction in naïve and resting memory cells at M2 (p < 0.01), concomitant to expansions of activated and TLM cells (p < 0.005). Most changes remained stable over the first year of infection with no significant differences between M2 and M12 after infection (**Figure 1**). Consistently, similar alterations remained in CHI-naïve, being partially reverted in CHI-ART individuals (**Figures 1C–J**).

**Figure 2:**
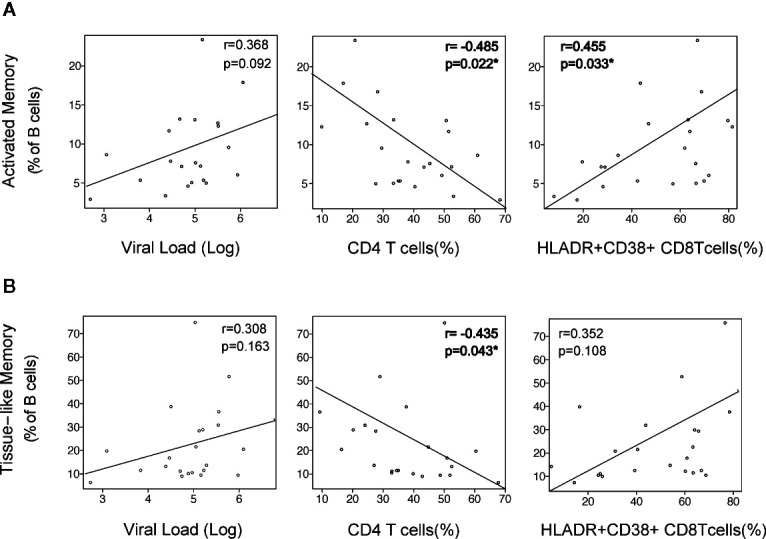
Association of B-cell activation with viral and immunological parameters. The alterations in Activated Memory **(A)** and TLM **(B)** B cells at month 2 after infection was correlated with virological and immunological parameters at the same timepoint: Plasma viral load (VL) as RNA Log10 copies/ml, CD4 depletion (frequency of CD4 T cells), and CD8 activation (frequency of HLA-DR+CD38+ CD8 T cells). Spearman r and p values are shown in each graph.

The initial alterations of B cell were associated with clinical and immunological parameters. The expansion of activated memory B cells observed at M2 negatively correlated with CD4 T-cell frequency (p = 0.022), positively correlated with CD8 T-cell activation (HLA-DR+CD38+ cells, p = 0.033), and showed a positive trend with VL that did not reach statistical significance. A similar observation was noticed for the early increase of TLM cells, although in this case only the negative association with CD4 T cells was significant (p = 0.043, **Figure 2**).

### Dynamics of PD-1 Expression

**Figure 3:**
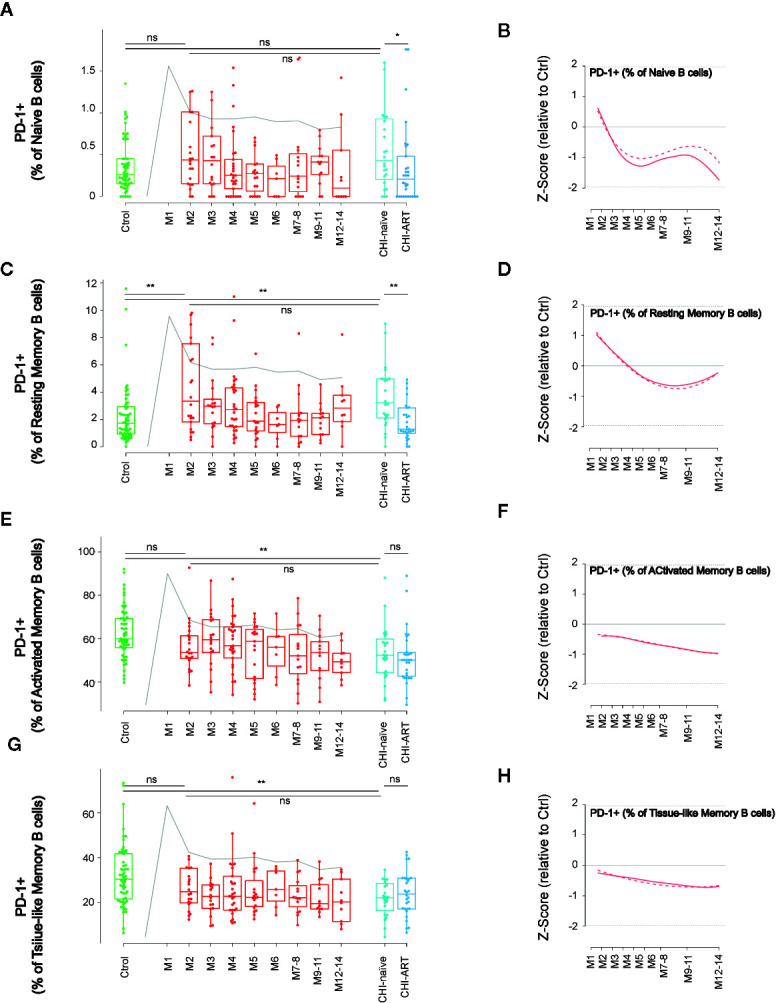
Dynamics of PD-1 expression in main B-cell subsets after HIV infection. As in **Figure 1**, left panels show individual data, median, IQR (box) and min/max values (bars, except for outliers) across the different timepoints and study groups: HIV uninfected (green), acutely HIV infected (red, M indicates month after primoinfection), and chronic untreated or treated HIV infected individuals (dark and light blue respectively). Gray line represents the profile of VL dynamics for reference. Right panels show the modeled dynamics as Z-score values for PHI individuals over HIV-uninfected individuals. Solid lines show non-parametric models, while dotted lines indicate the best fitting for polynomial time effects regression approximation. The frequency of PD-1+ cells in the indicated subsets defined by CD21 and CD27 markers **(A**–**H)** is shown. ns non significant; *p < 0.05; **p < 0.01.

The expression dynamics of PD-1 was characterized in the above-defined B-cell subsets. As compared to HIV-uninfected individuals, naïve and resting memory cells in PHI showed an early (M2) increase of PD-1 expression at M2 that was only significant for resting memory cells (p < 0.01), while this expression was normalized in CHI-ART (**Figures 3A–D**). In contrast, the PD-1 expression in activated and TLM B cells was not significantly different at M2 as compared to HIV-uninfected individuals, showing a progressive decrease during PHI. Also, the PD-1 expression in these two subsets was significantly lower in CHI-naïve (p < 0.01) and seemed to be not normalized in CHI-ART individuals, as compared to HIV-uninfected individuals (**Figures 3E–H**).

### Analysis of IgM/IgD Subsets

**Figure 4:**
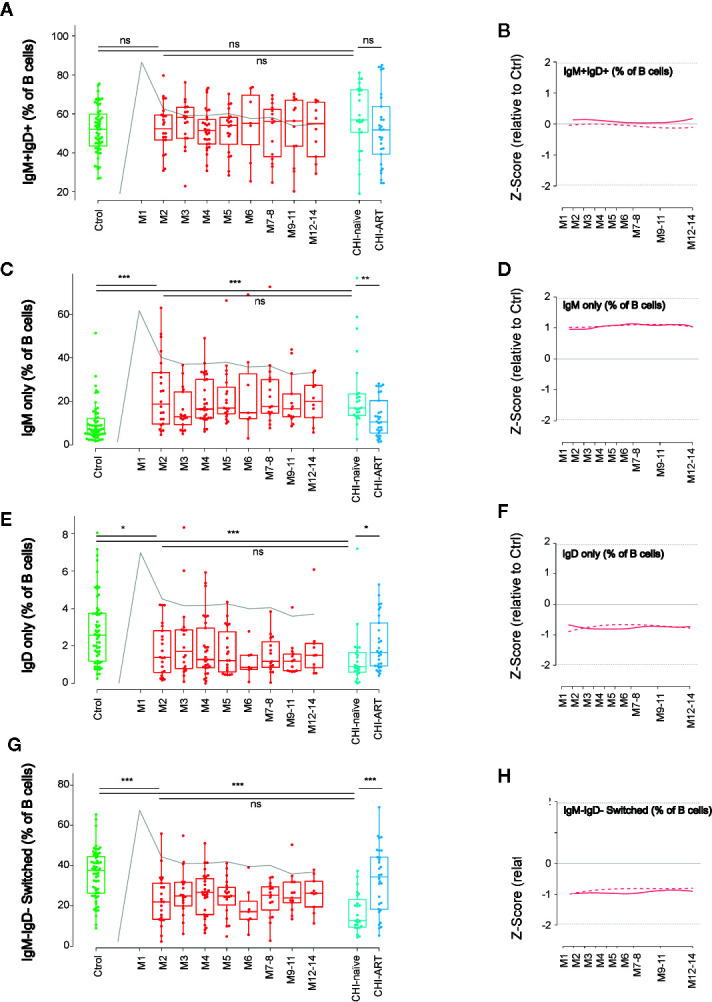
Early loss of switched B cells and IgD-only B cells. As in **Figure 1**, left panels show individual data, median, IQR (box), and min/max values (bars, except for outliers) across the different timepoints and study groups: HIV uninfected (green), acutely HIV infected (red, M indicates month after primoinfection), and chronic untreated or treated HIV infected individuals (dark and light blue respectively). Gray line represents the profile of VL dynamics for reference. Right panels show the modeled dynamics as Z-score values for PHI individuals over HIV-uninfected individuals. Solid lines show non-parametric models, while dotted lines indicate the best fitting for polynomial time effects regression approximation. The frequency of the different B-cell subsets defined by IgD and IgM expression **(A**–**H)** is shown. ns non significant; *p < 0.05; **p < 0.01; ***p < 0.005.

To further define the dynamics of memory B cells, we next analyzed the changes in B-cell subsets defined by the expression of IgM and IgD. The IgM+IgD+ subpopulation remained stable over PHI showing values similar to HIV-uninfected or CHI individuals (**Figures 4A, B**). In contrast, double negative switched, IgD-only and IgM-only B-cell subsets, were rapidly altered after HIV infection. The frequency of IgM-only B cells significantly increased at M2, as compared to HIV-uninfected individuals (p < 0.001 and p < 0.05, respectively), and remained stable over PHI and CHI, being partly recovered in CHI-ART (**Figures 4C, D**). An opposite behavior was observed for IgD-only and switched B cells, whose frequency significantly decreased after infection as compared to HIV-uninfected individuals (p < 0.05 and p < 0.001, respectively), and remained stable over PHI and CHI, being also recovered in CHI-ART individuals (**Figures 4E–H**).

### Dynamics of Transitional and Marginal Zone-Like B Cells

**Figure 5:**
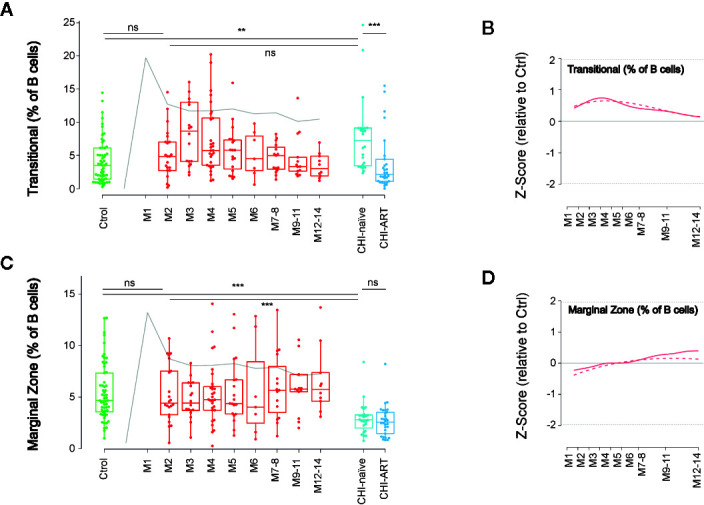
Late impact on Transitional and Marginal Zone-like B cells. As in **Figure 1**, left panels show individual data, median, IQR (box), and min/max values (bars, except for outliers) across the different timepoints and study groups: HIV uninfected (green), acutely HIV infected (red, M indicates month after primoinfection), and chronic untreated or treated HIV infected individuals (dark and light blue respectively). Gray line represents the profile of VL dynamics for reference. Right panels show the modeled dynamics as Z-score values for PHI individuals over HIV-uninfected individuals. Solid lines show non-parametric models, while dotted lines indicate the best fitting for polynomial time effects regression approximation. The frequency of Transitional B cells (CD21+CD10+CD38+CD27−, **A**, **B**) and Marginal Zone-like B cells (CD27+IgD+CD21+, **C**, **D**) is shown. ns non significant; **p < 0.01; ***p < 0.005.

In addition to the classical memory/naïve B-cell classification, we also explored the dynamics of other relevant subsets in B-cell function, known to be altered after HIV infection (7, 24). First, we analyzed the frequency of Transitional B cells, which are defined as CD19+CD10+CD38+CD27− cells (**Supplementary Figure 1**) and identify an intermediate state between immature and mature B cells. The frequency of Transitional B cells showed a complex behavior over PHI, with a significant increase after 3 months of HIV infection compared to M2 and uninfected individuals (p < 0.05 in both cases). This delayed expansion was followed by a stepwise return to values of uninfected individuals at M12. Despite this observation, transitional cells were significantly increased in CHI-naïve and normalized in CHI-ART, as compared to HIV-uninfected individuals (**Figures 5A, B**).

A second relevant subset is defined by CD19+CD27+IgD+CD21+ and identifies innate-like or MZ-like B cells, which are involved in T-cell independent B-cell responses. The frequency of MZ-like-B cells showed a unique behavior among B-cell subsets, with a remarkable stability over the first year of HIV infection and a significant decline in untreated CHI, as compared to HIV-uninfected individuals. Notably, this late depletion of MZ-like B cells seemed to be irreversible since it was not recovered in CHI-ART individuals (**Figures 5C, D**).

### Dynamics of Plasmablast Expansion and Association With Cytokine and Humoral Responses

**Figure 6:**
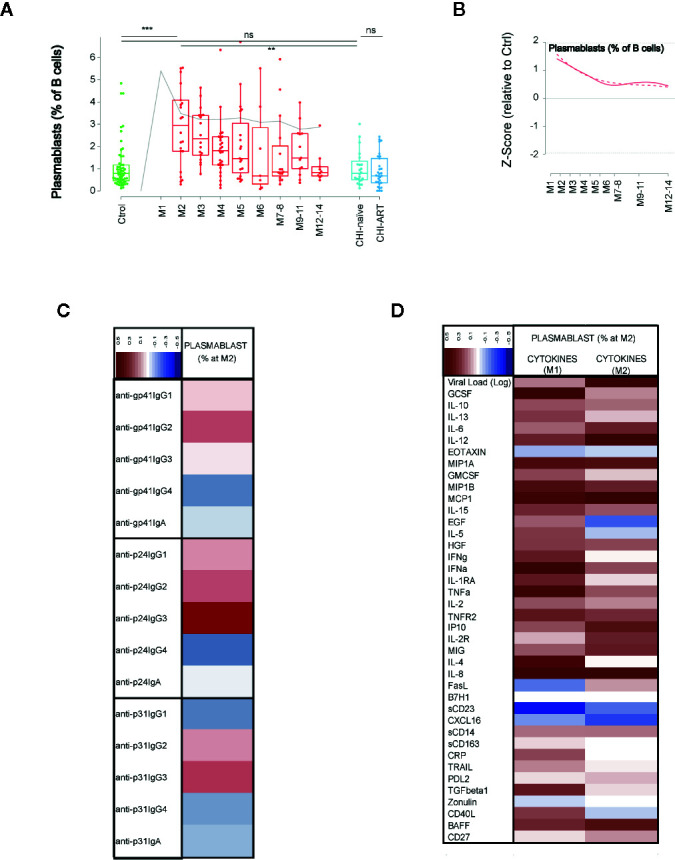
Dynamics of plasmablasts and association with immune responses. As in **Figure 1**, **(A)** shows individual data, median, IQR (box) and min/max values (bars, except for outliers) across the different timepoints and study groups: HIV uninfected (green), primary HIV infected (red, M indicates months after infection), and chronically naive and ART-treated HIV infected individuals (dark and light blue respectively). Gray line represents the profile of VL dynamics for clinical reference. **(B)** shows the modeled dynamics as Z-score values for PHI individuals over HIV-uninfected individuals. Solid lines show non-parametric models, while dotted lines indicate the best fitting for polynomial time effects regression approximation. **(C**, **D)** show the correlation of the plasmablasts’ frequency at month 2 after infection with the specific humoral responses against HIV proteins and with the levels of cytokines at 1 or 2 months after infection. Color code for correlation coefficients are shown in each panel (upper left corner). ns non significant; **p < 0.01; ***p < 0.005.

Finally, we addressed the dynamics of antibody secreting cells (plasmablast, CD38++CD27++ CD19+ cells). These cells showed an early significant expansion at M2 compared to uninfected individuals (p < 0.0005), expansion was transient and progressively declined until month 7–8, remaining stable afterwards (**Figures 6A, B**). To better describe the association of early plasmablast expansion and the elicitation of anti-HIV humoral responses, we analyzed the correlation of the frequency of plasmablasts at the peak (M2) with the IgG and IgA expression levels against three different viral antigens (the envelope glycoprotein subunit gp41, the gag Capsid protein p24, and the pol p31 integrase, **Figure 6C**). The IgG3 response against p24 showed the highest correlation with the plasmablast frequency (r = 0.54, p = 0.0085), while there was no significant association with any other of HIV-specific responses. Then, the association of plasmablast frequencies with the VL and cytokine levels was analyzed at M1 and M2 after infection. The frequency of plasmablasts positively correlated with VL at M2 (r = 0.513, p = 0.0146) after infection, but poorly with peak VL at M1 (r = 0.148, p = 0.509). From the wide range of analyzed cytokines, negative correlations were only significant for CXCL16 and sCD23 at M1 (r = −0.43, p = 0.0434), while the most significant positive correlation with plasmablast frequencies was found for GCSF at M1 (r = −0.57, p = 0.0054),. Additional significant positive correlations at M1 were observed for MCP-1, IFN-alpha, TNF-alpha, and IL-8 (p < 0.05). At M2, only IL-12, IL-8, and MCP-1 were positively associated with plasmablast frequencies (p < 0.05), being IL-12 the most significant (r = −0.55, p = 0.0087, **Figure 6D**).

## Discussion

We conducted a wide analysis of B-cell populations in peripheral blood of PHI individuals over the first year of infection. Our main objective was to characterize the dynamics of B-cell subsets during early HIV infection and evaluate their association with humoral and inflammatory responses.

Previous studies reported changes in the distribution of CD21and CD27 expression by B cells in chronic or early HIV infection (5, 8, 9). Our data indicate similar changes in AHI, mainly characterized by a decrease in naïve and resting memory cells and an increase in activated and TLM B cells. We additionally show that these changes are commonly sustained over the first year of HIV infection, and maintained in chronic untreated individuals. The early effect of HIV infection on these subsets shows some association with CD4 T-cell frequencies and activated CD8 T cells, suggesting that the earliest changes in B cells may be associated with the massive viral replication and CD4 T-cell depletion. In addition to these expected data, changes in PD-1 expression in these subsets were observed over the follow-up of PHI individuals. Lower PD-1 expression in Naïve and Resting memory B cells compared to Activated and TLM cells has been previously reported (25, 26), along with a partial decrease in PD-1 expressing memory cells in chronic infection (26). Consistently, our data indicate a gradual loss of PD-1 expression during PHI. This observation is particularly interesting, given the prominent role of PD-1 in B-cell depletion during acute HIV infection (27). However, the specific role of PD-1 may be modulated by the expression of a wide range of other inhibitory receptors (LAIR-1, CD22. CD32b, FCRL4, CD85), which are also perturbed by HIV infection (11), and may therefore contribute to the unresponsive state of B cells and to reduce the lifespan of these cells (28).

The analysis of IgM and IgD expression in B cells showed an increase in IgM only cells and a decrease in IgD only and switched (IgM-IgD-) cells, as previously described (8). These changes showed a stable kinetics over PHI and chronic infection with a slight recovery in individuals that initiated ART, suggesting again that early events in HIV infection lead to long-lasting immune perturbations. In contrast, Transitional and MZ-like B cells showed no significant differences at M2 early after HIV infection but later changes over the course of the infection, as compared to levels in HIV-uninfected individuals. Transitional B cells, a heterogeneous pool of pre-mature B cells (29), peaked at month 3–4 post infection and normalized afterwards, while levels were significantly increased in CHI-naïve individuals, as reported by others (6). Although homeostatic mechanisms could be behind this observation, it is unclear whether these mechanisms are triggered by rapid effects of HIV infection on B-cell survival or by the cytokine storm associated with PHI. Indeed, the frequency of Transitional B cells has been associated with plasma levels of IL-7 (30), a cytokine that is upregulated in acute (31) and chronic HIV infection. Unfortunately, we could not evaluate such an association in our cohort because IL-7 was not quantifiable in more than 75% of the samples and was therefore excluded from the analysis. A second particular dynamics was observed for MZ-like B cells. These cells are involved in T-cell independent responses in spleen (32) and its depletion in HIV infected individuals has been largely documented (7, 8, 33). However, our data show that MZ-like B cells remain stable over early infection, at least until 1 year, and they only irreversibly decline in chronic phases as compared to levels in HIV-uninfected individuals.

Finally, plasmablast dynamics showed early expansion until month 6 after infection (34). An in-depth analysis at the peak of plasmablast expansion showed a positive correlation with the levels of IgG3 anti-p24 antibodies, suggesting a predominant response to this antigen. Consistently, previous studies have shown that HIV-specific IgG3 responses, in particular against p55Gag, peak during acute HIV infection (22, 35). In addition, levels of some cytokines showed certain association with plasmablast expansion. While positive associations were observed for most inflammatory cytokines, in particular IL-12, which is involved in B-cell differentiation (36), this could be explained by the parallel association with VL, suggesting antigen driven expansion. Of particular interest is the negative association observed for the levels of sCD23 and to a lesser extent CXCL6. Previous data in this cohort showed that plasma sCD23 levels were significantly decreased prior to seroconversion (22), which could explain the negative correlation. However, the functional implications are unclear and maybe related to the complex modulation of Th2 responses in which IgE and its receptor CD23 have been recently involved (37).

Although our study provides new insights on the dynamics of B cells during PHI, it has several limitations. First, due to study design, no data on B-cell subsets was available at the peak of viremia (M1), compromising the identification of earliest B-cell changes, such as the peak of plasmablast expansion, reported at day 7 post infection (17) or the rapid changes in PD-1 expression (27). Furthermore, differences found in the CHI groups could be influenced by older age and the national guidelines at the moment of the study (2013–2014) that recommended ART initiation in patients with CD4 T-cell counts ≤350 cells/mm^3^ or presenting with an AIDS-defining event. Late ART initiation and age are known to impact immune status and immune recovery in HIV infected individuals (38, 39), thus potentially biasing the naïve and ART-treated CHI groups. A final limitation is associated with the loss to follow-up of PHI individuals. Although common in rural settings (56), low retention rate was further affected in our study by migration and pregnancy.

In conclusion, our data suggest that the effects of HIV infection on B cells occur in different waves. Early changes involve B-cell activation and are maintained over early and chronic infection. Transient changes were observed in plasmablasts and transitional B cells (peaking at month 2 and month 3–4, respectively), while a late wave of effects impact specifically MZ-like B cells, which are stable over the first year of infection but drop in chronic infection. Our data also showed complex dynamics of check point receptor PD-1, whose changes extend beyond virological and immunological setpoint (occurring at M3 and 6, respectively). Further detrimental events for B cells were associated with sustained viral replication, in particular MZ-like B-cell depletion and progressive damage of resting and switched memory B cells. Therefore, we postulate that several waves of cytopathic events affect B cells over the course of HIV infection, leading to the impaired responses to vaccines or antigens (either T-dependent or independent) in chronic infection. These observations further highlight the need for early ART initiation, which is known to minimize immune B-cell damage and recover B-cell function (40, 41).

## Data Availability Statement

The raw data supporting the conclusions of this article will be made available by the authors, without undue reservation.

## Ethics Statement

The studies involving human participants were reviewed and approved by the Ministry of Health of Mozambique (461/CNBS/12). The patients/participants provided their written informed consent to participate in this study.

## Author Contributions

DN, JC, and JB designed the study and the analysis. LP, EP, LF-S, IM, and CJ performed laboratory analysis. LP, MJ, VU, and JC performed PBMCs phenotyping and validation of the data. MJ and VU performed statistical analyses. MJ, VU, JC, DN, and JB interpreted the data. MJ and JB drafted the paper. MJ, LP, VU, DN, and JC provided relevant critical data review and reviewed the manuscript writing. All authors contributed to the article and approved the submitted version.

## Funding

This work was supported by The Spanish Ministry of Science (Mineco) (SAF-2011-27901) to DN; Bill and Melinda Gates Foundation (OPP1068252) to DN and The Spanish Ministry of Health through the Institute of Health Carlos III (ISCIII) (FI12/00096) to LP and (DTS15/00185) to JB. The funders had no role in study design, data collection and analysis, decision to publish, or preparation of the manuscript.

## Conflict of Interest

Unrelated to this work, JB is CEO and founder of AlbaJuna Therapeutics, S.L. and JC is CSO and founder of AlbaJuna Therapeutics, S.L.

The remaining authors declare that the research was conducted in the absence of any direct commercial or financial relationships that could be construed as a potential conflict of interest.

## References

[1] McCune JM The dynamics of CD4+ T-cell depletion in HIV disease. Nature (2001) 410:974–9. 10.1038/35073648 11309627

[2] Moir S Chun T-WW Fauci AS Pathogenic mechanisms of HIV disease. Annu Rev Pathol (2011) 6:223–48. 10.1146/annurev-pathol-011110-130254 21034222

[3] Deeks SG HIV infection, inflammation, immunosenescence, and aging. Annu Rev Med (2011) 62:141–55. 10.1146/annurev-med-042909-093756 PMC3759035 21090961

[4] De Milito A Nilsson A Titanji K Thorstensson R Reizenstein E Narita M Mechanisms of hypergammaglobulinemia and impaired antigen-specific humoral immunity in HIV-1 infection. Blood (2004) 103:2180–6. 10.1182/blood-2003-07-2375 14604962

[5] Moir S Fauci AS B-cell responses to HIV infection. Immunol Rev (2017) 275:33–48. 10.1111/imr.12502 28133792 PMC5300048

[6] Malaspina A Moir S Ho J Wang W Howell ML O’Shea MA Appearance of immature/transitional B cells in HIV-infected individuals with advanced disease: correlation with increased IL-7. Proc Natl Acad Sci USA (2006) 103:2262–7. 10.1073/pnas.0511094103 PMC1413756 16461915

[7] Liechti T Kadelka C Braun DL Kuster H Boni J Robbiani M Widespread B cell perturbations in HIV-1 infection afflict naive and marginal zone B cells. J Exp Med (2019) 9:2071–90. 10.1084/jem.20181124 PMC6719425 31221742

[8] Carrillo J Negredo E Puig J Molinos-Albert LM Rodríguez de la Concepción ML Curriu M Memory B cell dysregulation in HIV-1-infected individuals. AIDS (2018) 32:149–60. 10.1097/QAD.0000000000001686 29112067

[9] Moir S Ho J Malaspina A Wang W DiPoto AC O’Shea MA Evidence for HIV-associated B cell exhaustion in a dysfunctional memory B cell compartment in HIV-infected viremic individuals. J Exp Med (2008) 205:1797–805. 10.1084/jem.20072683 PMC2525604 18625747

[10] Boliar S Murphy MK Tran TC Carnathan DG Armstrong WS Silvestri G B-Lymphocyte Dysfunction in Chronic HIV-1 Infection Does Not Prevent Cross-Clade Neutralization Breadth. J Virol (2012) 86:8031–40. 10.1128/jvi.00771-12 PMC3421653 22623771

[11] Moir S Fauci AS B-cell exhaustion in HIV infection: The role of immune activation. Curr Opin HIV AIDS (2014) 9:472–7. 10.1097/COH.0000000000000092 25023621

[12] Kernéis S Launay O Turbelin C Batteux F Hanslik T Boëlle PY Long-term immune responses to vaccination in HIV-infected patients: A systematic review and meta-analysis. Clin Infect Dis (2014). 10.1093/cid/cit937 PMC4761378 24415637

[13] Kardava L Moir S Shah N Wang W Wilson R Buckner CM Abnormal B cell memory subsets dominate HIV-specific responses in infected individuals. J Clin Invest (2014) 124:3252–62. 10.1172/JCI74351 PMC4071400 24892810

[14] Robb ML Eller LA Kibuuka H Rono K Maganga L Nitayaphan S Prospective Study of Acute HIV-1 Infection in Adults in East Africa and Thailand. N Engl J Med (2016) 374:2120–30. 10.1056/NEJMoa1508952 PMC5111628 27192360

[15] Pastor L Urrea V Carrillo J Parker E Fuente-Soro L Jairoce C Dynamics of CD4 and CD8 T-Cell Subsets and Inflammatory Biomarkers during Early and Chronic HIV Infection in Mozambican Adults. Front Immunol (2017) 8:1925. 10.3389/fimmu.2017.01925 29354131 PMC5760549

[16] Kazer SW Aicher TP Muema DM Carroll SL Ordovas-Montanes J Miao VN Integrated single-cell analysis of multicellular immune dynamics during hyperacute HIV-1 infection. Nat Med (2020) 26:511–8. 10.1038/s41591-020-0799-2 PMC7237067 32251406

[17] Mabuka JM Dugast A-S Muema DM Reddy T Ramlakhan Y Euler Z Plasma CXCL13 but Not B Cell Frequencies in Acute HIV Infection Predicts Emergence of Cross-Neutralizing Antibodies. Front Immunol (2017) 8:1104. 10.3389/fimmu.2017.01104 28943879 PMC5596076

[18] Cagigi A Nilsson A Pensieroso S Chiodi F Dysfunctional B-cell responses during HIV-1 infection: implication for influenza vaccination and highly active antiretroviral therapy. Lancet Infect Dis (2010) 10:499–503. 10.1016/S1473-3099(10)70117-1 20610332

[19] Titanji K De Milito A Cagigi A Thorstensson R Grützmeier S Atlas A Loss of memory B cells impairs maintenance of long-term serologic memory during HIV-1 infection. Blood (2006) 108:1580–7. 10.1182/blood-2005-11-013383 16645169

[20] Farooq PD Sherman KE Hepatitis B Vaccination and Waning Hepatitis B Immunity in Persons Living with HIV. Curr HIV/AIDS Rep (2019) 16:395–403. 10.1007/s11904-019-00461-6 31468298

[21] Id P Langhorst J Schro D Ruffer A Carrillo J Urrea V Different pattern of stool and plasma gastrointestinal damage biomarkers during primary and chronic HIV infection. PLoS One (2019) 14:e0218000. 10.1371/journal.pone.0218000 31185037 PMC6559643

[22] Pastor L Parker E Carrillo J Urrea V Fuente-Soro L Respeito D A Cytokine Pattern That Differentiates Preseroconversion From Postseroconversion Phases of Primary HIV Infection. J Acquir Immune Defic Syndr (2017) 74:459–66. 10.1097/QAI.0000000000001272 28225519

[23] Pastor L Casellas A Carrillo J Alonso S Parker E Fuente-Soro L IP-10 Levels as an Accurate Screening Tool to Detect Acute HIV Infection in Resource-Limited Settings. Sci Rep (2017) 7:8104. 10.1038/s41598-017-08218-0 28808319 PMC5556096

[24] Amu S Fievez V Nozza S Lopalco L Chiodi F Dysfunctions in the migratory phenotype and properties of circulating immature transitional B cells during HIV-1 infection. AIDS (2016) 30:2169–77. 10.1097/QAD.0000000000001182 27281060

[25] Rethi B Sammicheli S Amu S Pensieroso S Hejdeman B Schepis D Concerted effect of lymphopenia, viraemia and T-cell activation on Fas expression of peripheral B cells in HIV-1-infected patients. AIDS (2013) 27:155–62. 10.1097/QAD.0b013e32835b8c5e 23238551

[26] Amu S Lavy-Shahaf G Cagigi A Hejdeman B Nozza S Lopalco L Frequency and phenotype of B cell subpopulations in young and aged HIV-1 infected patients receiving ART. Retrovirology (2014) 11:76. 10.1186/s12977-014-0076-x 25213015 PMC4172851

[27] Titanji K Velu V Chennareddi L Vijay-Kumar M Gewirtz AT Freeman GJ Acute depletion of activated memory B cells involves the PD-1 pathway in rapidly progressing SIV-infected macaques. J Clin Invest (2010) 120:3878–90. 10.1172/JCI43271 PMC2964982 20972331

[28] Samuelsson A Broström C van Dijk N Sönnerborg A Chiodi F Apoptosis of CD4+ and CD19+ cells during human immunodeficiency virus type 1 infection–correlation with clinical progression, viral load, and loss of humoral immunity. Virology (1997) 238:180–8. 10.1006/viro.1997.8790 9400591

[29] Chung JB Silverman M Monroe JG Transitional B cells: Step by step towards immune competence. Trends Immunol (2003) 24:342–8. 10.1016/S1471-4906(03)00119-4 12810111

[30] Malaspina A Moir S Chaitt DG Rehm CA Kottilil S Falloon J Idiopathic CD4+ T lymphocytopenia is associated with increases in immature/transitional B cells and serum levels of IL-7. Blood (2007) 109:2086–8. 10.1182/blood-2006-06-031385 PMC1801046 17053062

[31] Roberts L Passmore J-ASA Williamson C Little F Bebell LM Mlisana K Plasma cytokine levels during acute HIV-1 infection predict HIV disease progression. AIDS (2010) 24:819–31. 10.1097/QAD.0b013e3283367836 PMC3001189 20224308

[32] Cerutti A Cols M Puga I Marginal zone B cells: Virtues of innate-like antibody-producing lymphocytes. Nat Rev Immunol (2013) 13:118–32. 10.1038/nri3383 PMC3652659 23348416

[33] Demberg T Mohanram V Musich T Brocca-Cofano E McKinnon KM Venzon D Loss of marginal zone B-cells in SHIVSF162P4 challenged rhesus macaques despite control of viremia to low or undetectable levels in chronic infection. Virology (2015) 484:323–33. 10.1016/j.virol.2015.06.022 PMC4567457 26151223

[34] Fink K Origin and Function of Circulating Plasmablasts during Acute Viral Infections. Front Immunol (2012) 3:78. 10.3389/fimmu.2012.00078 22566959 PMC3341968

[35] Yates NL Lucas JT Nolen TL Vandergrift NA Soderberg KA Seaton KE Multiple HIV-1-specific IgG3 responses decline during acute HIV-1. AIDS (2011) 25:2089–97. 10.1097/qad.0b013e32834b348e PMC3667583 21832938

[36] Moens L Tangye SG Cytokine-mediated regulation of plasma cell generation: IL-21 takes center stage. Front Immunol (2014) 5:65. 10.3389/fimmu.2014.00065 24600453 PMC3927127

[37] Shan M Carrillo J Yeste A Gutzeit C Segura-Garzón D Walland AC Secreted IgD Amplifies Humoral T Helper 2 Cell Responses by Binding Basophils via Galectin-9 and CD44. Immunity (2018) 49:709–24.e8. 10.1016/j.immuni.2018.08.013 30291028 PMC6366614

[38] Negredo E Back D Blanco J-R Blanco J Erlandson KM Garolera M Aging in HIV-Infected Subjects: A New Scenario and a New View. BioMed Res Int (2017) 2017:5897298. 10.1155/2017/5897298 29430462 PMC5753008

[39] Massanella M Negredo E Clotet B Blanco J Immunodiscordant responses to HAART–mechanisms and consequences. Expert Rev Clin Immunol (2013) 9:1135–49. 10.1586/1744666X.2013.842897 24168417

[40] Moir S Buckner CM Ho J Wang W Chen J Waldner AJ B cells in early and chronic HIV infection: Evidence for preservation of immune function associated with early initiation of antiretroviral therapy. Blood (2010) 116:5571–9. 10.1182/blood-2010-05-285528 PMC3031405 20837780

[41] Pensieroso S Cagigi A Palma P Nilsson A Capponi C Freda E Timing of HAART defines the integrity of memory B cells and the longevity of humoral responses in HIV-1 vertically-infected children. Proc Natl Acad Sci USA (2009) 106:7939–44. 10.1073/pnas.0901702106 PMC2683072 19416836

