# Supplementary material for: A Longitudinal Analysis Reveals Early Activation and Late Alterations in B Cells During Primary HIV Infection in Mozambican Adults
Source: Front Immunol. 2021 Jan 15;11:614319. doi: 10.3389/fimmu.2020.614319 (PMC7844141; doi:10.3389/fimmu.2020.614319)
Supplement: Supplementary file 1 [file DataSheet_1.docx]

Supplementary Material

A longitudinal analysis reveals early activation and late alterations in B cells during acute HIV infection.

Montse Jiménez, Lucia Pastor, Victor Urrea, Erica Parker, Laura Fuente-Soro, Chenjerai Jairoce, Inacio Mandomando, Jorge Carrillo, Denise Naniche, Julià Blanco

*** Correspondence:**

Julià Blanco
jblanco@irsicaixa.es

# Supplementary Figure 1. Gating strategy for B-cell analysis.

**Supplementary Figure 1. Gating strategy for B cell phenotype.** Acquisition was controlled over time and singlets were gated to identify lymphocytes. Then, living cells were identified by viability staining and B cells defined by CD21 and CD19 expression (CD19+ CD21+/- cells were gated). Mature B cells were identified by excluding transitional and plasma cells in a CD38/CD10 dot plot and analyzed for maturation status in a CD21/CD27 dot plot to define Naïve and Resting, Activated and Tissue-like memory cells. CD38/CD27 markers were used to identify plasmablast and CD10/CD38 to identify transitional cells (further refined by CD27 negativity. Marginal Zone-like B cells were defined ans CD27+IgD+ cells. IgD, IgM and PD-1 expression was also analyzed.

# Table 1. Clinical and demographic characteristics of study population according to HIV-status.

|  | **HIV+ PHI with follow-up (n=40)** | **HIV-uninfected (n=58)** | **CHI-naïve**  **(n=26)** | **CHI-ART**  **(n=30)** | **P-value** |
| --- | --- | --- | --- | --- | --- |
| **Age (years)** [Mean (SD)] | 27.2 (9.2) | 27.9 (9.5) | 38.2 (13.4) | 42.9 (8.8) | **0.0001^a^** |
| **Gender** [Female (%)] | 24 (60.0%) | 46 (79.3%) | 19 (73.1%) | 19 (63.3%) | 0.162^b^ |
| **BMI (kg/m2)** [Mean (SD)] | 20.3 (3.1) | 21.5 (4.1) | 24.5 (4.6) | 24.1 (3.2) | **0.0001^a^** |
| **time on ART (years)** [Median (IQR)] | - | - | - | 2.6 (0.9-4.5) | - |
| **Pregnant** [n (% F)] | 3 (12.5%) | 7 (15.2%) | 0 (0%) | 3 (15.8%) | 0.348^b^ |
| **Fever last 24h**  [n (%)] | 5 (12.5%) | 3 (5.3%) | 4 (15.4%) | 1 (3.3%) | 0.246^b^ |
| **Intestinal complaint last week** [n (%)] | 12 (30%) | 15 (25.9%) | 4 (15.4%) | 2 (6.7%) | 0.067^b^ |

^a^ Comparisons of continuous variables were performed by Kruskal Wallis test.

^b^ Comparisons for proportions were performed by Fisher exact test.
